# Role of the Skin Immune System in Wound Healing

**DOI:** 10.3390/cells13070624

**Published:** 2024-04-04

**Authors:** Angela Cioce, Andrea Cavani, Caterina Cattani, Fernanda Scopelliti

**Affiliations:** National Institute for Health, Migration and Poverty INMP/NIHMP, Via di S.Gallicano, 25, 00153 Rome, Italy; angela.cioce@inmp.it (A.C.); andrea.cavani@inmp.it (A.C.); caterina.cattani@inmp.it (C.C.)

**Keywords:** skin, immune system, wound healing, wound repair

## Abstract

Wound healing is a dynamic and complex process, characterized by the coordinated activities of multiple cell types, each with distinct roles in the stages of hemostasis, inflammation, proliferation, and remodeling. The cells of the immune system not only act as sentinels to monitor the skin and promote homeostasis, but they also play an important role in the process of skin wound repair. Skin-resident and recruited immune cells release cytokines and growth factors that promote the amplification of the inflammatory process. They also work with non-immune cells to remove invading pathogens and debris, as well as guide the regeneration of damaged host tissues. Dysregulation of the immune system at any stage of the process may lead to a prolongation of the inflammatory phase and the development of a pathological condition, such as a chronic wound. The present review aims to summarize the roles of different immune cells, with special emphasis on the different stages of the wound healing process.

## 1. Wound Healing Overview

Wound healing involves a series of coordinated and overlapping cellular events and is a complex and dynamic process. It is classically simplified into the following four main phases: hemostasis, inflammatory response, proliferation, and dermal remodeling [1,2]. Hemostasis, the first step in the wound healing process, stops bleeding after vascular damage. Hemostasis begins with the vasoconstriction of vessel walls, followed by platelet aggregation and platelet plug formation (primary hemostasis). Next, the coagulation cascade is activated, converting soluble fibrinogen into insoluble filaments that form the fibrin network (secondary hemostasis). The platelet cap and fibrin network join together to form the thrombus, which stops the blood flow [3]. The inflammatory phase is typified by the strong involvement of resident and recruited cells of the innate and adaptive immune system. This phase is concomitant with hemostasis and aims to clean wounds of pathogens and debris. Following injury, resident skin cells are exposed to danger signals, like pathogen-associated molecular patterns (PAMPs) and danger-associated molecular patterns (DAMPs) [4]. Damaged cells at the wound edge also release endogenous molecules that act as DAMPs, which are recognized by cellular pattern recognition receptors (PRRs), such as Toll-Like Receptors (TLRs), and provide danger signals aimed at alerting the immune system [5]. DAMPs include DNA debris, as well as cytoplasmic molecules released by dead cells. The interaction of TLRs with PAMPs or DAMPs activates several intracellular signaling pathways that produce inflammatory cytokines, such as TNF-α and IL-1; antimicrobial molecules; and chemokines, such as CXCL1, CXCL5, and CXCL8 [6]. Two important pathways activated as a consequence of skin barrier disruption are p44/42 MAPK and p38 MAPK, which initiate a cascade of signaling pathways involved in the early phases of the healing process [7]. Another pathway activated is that of the SAPK/JNK kinase, which plays an important role in the regulation of pro-inflammatory responses. By phosphorylating a number of signaling molecules, it regulates gene expression and survival, as well as cellular metabolism [8]. After injury, keratinocytes also release pro-inflammatory cytokines that stimulate the migration and proliferation of neutrophils and macrophages at the wound site, such as TNF-α, IL-1, IL-6, and IL-8 [9]. In a normal skin wound healing process, inflammation usually lasts for 2–5 days and ends once the harmful stimuli are removed [10].

As the inflammation recedes, the proliferation phase begins. This phase is characterized by the re-epithelialization of the wound, the development of new blood vessels, and the formation of granulation tissue, which consists of large numbers of fibroblasts, granulocytes, macrophages, blood vessels, and collagen bundles and partially reconstitutes the structure and function of the damaged skin. Signals such as nitric oxide, cytokines, and growth factors, released by several cell types at the site of injury, stimulate re-epithelialization. During this phase, keratinocytes at the wound edges and epithelial stem cells from hair follicles begin to proliferate and migrate to cover the wound. The migration process stops when cells contact each other and form new adhesion structures [11]. This results in a thin epithelial layer that covers the wound [12]. Additionally, growth factors such as vascular endothelial growth factor (VEGF-A), platelet-derived growth factor (PDGF), and basic fibroblast growth factor (bFGF) stimulate angiogenesis. At this stage, endothelial cells proliferate to form new vessels that can deliver oxygen and nutrients to the damaged site [13]. In addition to their reactivity to growth factors, endothelial cells have other receptors that promote angiogenesis. In case of injury, endothelial cells express receptors on their surface, such as P-selectin, E-selectin and adhesion molecules ICAM-1 and VCAM-1, that promote adhesion and the infiltration of leukocytes at the damaged site. Studies have shown that deletion of P-selectin, E-selectin, ICAM-1, or VCAM-1 inhibited both neovascularization and wound healing, thus stressing the importance of endothelial cell–leukocyte interactions during skin repair [14,15].

Remodeling is the last step of the healing process, leading to wound maturation. During this phase, the neo-vascularization regresses and the granulation tissue is replaced by scar tissue. Granulation tissue is composed mainly of type III collagen, which is then replaced by type I collagen, the primary component of healthy skin [3]. This process is achieved through a balance between the synthesis of type I collagen and the degradation of type III collagen, which results in ECM remodeling [16].

Both resident and recruited immune cells have active functions in the repair of skin wounds. The present review aims to summarize the roles of the key players of the immune system in the different stages of the wound healing process.

## 2. Skin Immune System Overview

The skin is the largest organ of the human body and serves as the first line of defense against infection. Its main functions include protecting the host from danger signals through physical barriers, the release of biomolecules, and the interaction between the resident and recruited cells that constitute the so-called skin immune system [17,18].

**Figure 1:**
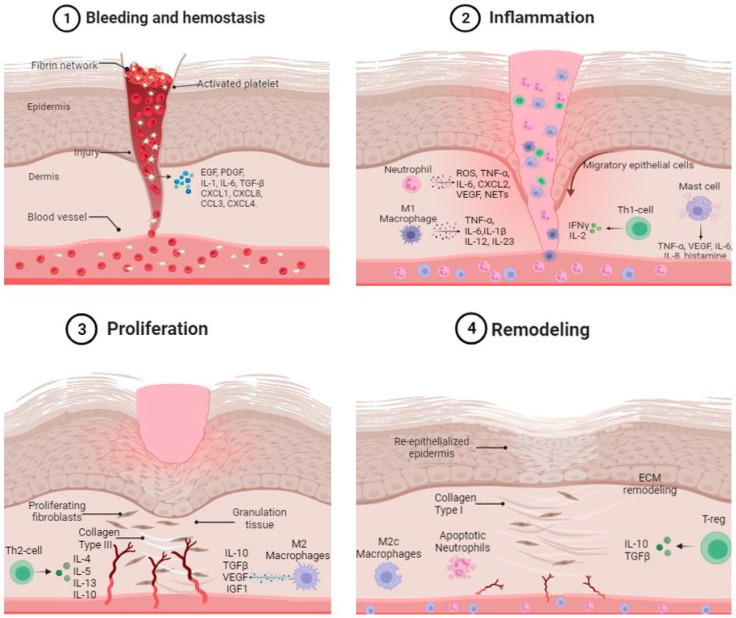
Representation of the roles of skin immune system cells in the different stages of the wound healing.

The skin consists of the following two distinct structural layers: the epidermis and the dermis [17]. The epidermis is a stratified, keratinized epithelium, the outermost layer of which consists of corneocytes that are cemented together by intercorneocyte lipids. This layer serves as a physical barrier, preventing the entry of chemicals and pathogens [19]. The dermis is a highly vascularized tissue containing hair follicles, nerve endings, and secretory glands. It is composed of fibroblasts, which produce extracellular matrix (ECM) proteins that give the skin its elasticity and strength [20]. Antimicrobial peptides (AMPs), such as defensins, are acidic in PH and contribute to the skin’s protective functions, because they make the skin an inhospitable environment for potential pathogens [21]. Importantly, the skin contains both resident and recruited immune cells, including mast cells, dendritic cells (DCs), lymphocytes, and macrophages. Resident and recruited cells form the skin immune system, which acts as an integrated sentinel device, constantly monitoring the skin and promoting the maintenance of homeostasis [22]. In case of injuries, immune cells from peripheral blood are recruited to the damaged area and interact with resident cells, thus activating a cascade of events intended to eliminate the insult and repair the wound. Keratinocytes and melanocytes, along with recruited immune cells, play crucial roles in establishing an adequate immune response against potential pathogens. This occurs both in the early stages after skin injury and later on, participating in the amplification of the inflammatory response. The bacteria that colonize wounds belong mainly to the families of Staphylococcaceae and Pseudomonaceae. In particular, Gram-negative bacteria belonging to the Staphylococcus genera are the first to invade wounds, followed by Gram-negative bacteria, such as *Pseudomonas* spp., *E. coli*, *Klebsiella pneumoniae* and *Enterobacter* spp. Once in the wound, these bacteria release microbial products that promote their survival and persistence in the host. It has been observed that their presence contributes to the prolongation of the inflammatory phase, the development of infection, and delays in the healing process. Therefore, it is essential to prevent the entry of these pathogens or clean the wound of them for successful wound closure [23]. Keratinocytes have TLRs, which recognize the PAMPs typical of pathogenic organisms. Once triggered, TLRs activate second messengers, such as inflammasomes and NFkB proteins. Moreover, keratinocytes release various pro-inflammatory cytokines, such as IL-1, IL-6, INF-γ, and TNF-α, as well as chemokines, such as CCL27, which are essential for the activation and recruitment of immune system cells [24]. Melanocytes also express a variety of TLRs, including TLR1, TLR2, and TLR6. Melanocytes regulate the immune response by releasing cytokines, such as IL-figβ, IL-6, TNF-α, and chemokines, such as CCL2 and CCL3, which are involved in leukocyte recruitment. Additionally, melanocytes promote the phagocytosis of pathogens [25] (Figure 1).

## 3. Role of Skin Immune System in Hemostasis

### 3.1. Platelets

The platelet is the main cell involved in this process. Platelets are nucleated cells that are derived from megakaryocytes, do not interact with endothelial cells, and circulate preferentially near the vessel wall under physiological conditions. In fact, the integrity of the endothelial coating provides a barrier that prevents platelet activation and aggregation. This is due, in part, to the release of substances such as nitric oxide and prostacyclins [26].

Following tissue injury and microcirculation disruption, damaged cells release vasoconstrictors, such as endothelin, to reduce bleeding. Vasoconstriction is also regulated by circulating catecholamines, epinephrine, norepinephrine, and prostaglandins [3]. Simultaneously, platelets adhere to the damaged endothelium through the interaction between the platelet receptor GPIb-IX-V and the collagen-bound von Willebrand factor (vWF). Hemostasis initiates platelet activation, leading to the generation of thrombin, which contributes to the formation of a hemostatic plug. In particular, thrombin binds to PAR1 and PAR4 receptors [27], stimulating platelets.

### 3.2. Platelets and Inflammation

In addition to their well-characterized role in hemostasis, platelets play important roles in immunity and inflammation. Indeed, in the hemostatic plug, activated platelets release a plethora of cytokines and chemokines that promote the recruitment of immune system cells and regulate subsequent steps in the healing process [28]. In particular, platelets have three distinct types of cytoplasmic granules, containing more than 300 molecules, including platelet factor 4 (PF4), epidermal growth factor (EGF), platelet-derived growth factor (PDGF), IL-1, IL-6, and TGF-β, as well as chemokines, such as CXCL1, CXCL8, CCL3 CXCL4, CXCL5, and CXCL7 [29]. Platelet factor 4 is a chemotactic factor for neutrophils, monocytes, and fibroblasts, and it promotes the differentiation of monocytes into macrophages [30]. PDGF leads to the induction of chemotaxis and the proliferation of immune cells, promoting the formation of new blood vessels and granulation tissue [31]. TGF-β1 stimulates keratinocyte proliferation, as well as the remodeling and regeneration of the epidermal layer [32]. IL-1 and IL-6 activate the complement cascade and C5, increasing vascular permeability and stimulating the migration of neutrophils and macrophages [33,34].

The chemokine CXCL4 promotes neutrophil activation, including adhesion and degranulation, and contributes to the differentiation of monocytes recruited to the site of skin injury into macrophages [35]. Additionally, CXCL4 and CCL5 increase the adhesiveness of circulating monocytes to the endothelium of the dermal microvasculature [36]. CXCL7 is the most abundant platelet-derived chemokine. Upon proteolytic cleavage, CXCL7 is cleaved into the following four chemokines: PBP, CTAP-III, β-TG, and NAP-2. However, only NAP-2 has chemotactic activity [37]. Releasing pro-inflammatory molecules, platelets are indeed the initiators of the inflammatory phase of tissue repair. Hemostatic plug formation and immune cell recruitment, mediated by activated platelets, prevent the invasion of microbial agents into the bloodstream. In addition, recent studies suggest that platelets also play a role in linking innate and adaptive immune responses. Activated platelets express intracellular MHC class I molecules on their surface and, together with other molecules required for synapse formation with T cells, such as the costimulatory molecule CD86, could present antigens and promote T-cell activation [38]. In the case of viral infection, mouse platelets were shown to modulate adaptive immune responses both in vivo and in vitro. The immune response to adenovirus was attenuated in platelet-depleted mice, but improvements in humoral and cellular responses were observed following platelet infusion [39]. Accordingly, in vitro studies have indicated that platelet-derived biomolecules, such as platelet lysate (PL), promote tissue repair and facilitate the process of wound healing by stimulating macrophage polarization in the M2-like phenotype. In fact, platelet lysate reduced the expression of costimulatory molecules CD80 and CD86, decreased secretion of the pro-inflammatory cytokine TNF-α, and increased secretion of TGF-β, a cytokine involved in the regenerative phase of the healing process [40].

Additionally, platelet derivatives could induce a transient increase in Th1 cytokines, followed by an expansion of TGF-β^+^ T regulatory cells, which promote tissue regeneration [41].

## 4. Role of the Innate and Adaptive Immune Cells in the Inflammatory and Proliferative Phases of Wound Healing

### 4.1. Neutrophils

Neutrophils are the first immune system cells to migrate to the injury site, and they remain the most abundant cell type in the first 24 h. Their primary function is to degrade and eliminate pathogens by releasing antimicrobial substances, reactive oxygen species (ROS), and proteases, contained in cytoplasmic granules [33]. During the neutrophil maturation process, various granules are formed, including azurophil granules (primary granules) and secondary granules. The primary granules are rich in peroxidase, azurocidin, lysozyme, and defensins, while the secondary granules are rich in lactoferrin, as well as metalloproteases such as MMP-8 and cathelicidin (hCAP-18), an antimicrobial protein. Due to the functional diversity of granular substances, neutrophils target pathogens through different mechanisms. For example, defensins act by creating pores on bacterial membranes. Lactoferrin interacts with iron-dependent metabolic pathways, while azurocidin enhances bacterial wall permeability [42].

**Figure 2:**
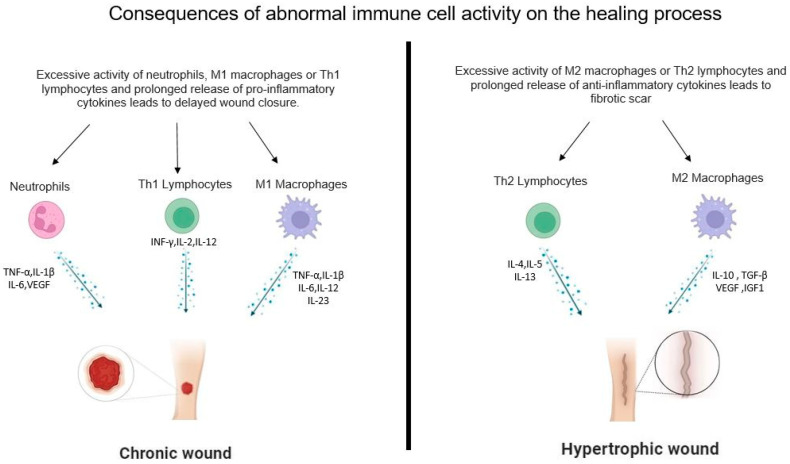
Consequences of abnormal immune cell activity on the healing process.

MMPs function by degrading extracellular matrix components and facilitating the migration of immune system cells from circulation to the site of injury. Although MMPs play a critical role in the repair process, several clinical studies indicate that overproduction of metalloproteases increases damage at the lesion site, leading to prolonged inflammation and impaired wound healing [43]. Neutrophils strengthen their antimicrobial capabilities by releasing neutrophil extracellular traps (NETs), which consist of nuclear chromatin, decorated with proteins, typically confined to their granules. The cationic component of NETs enables electrostatic bonding with the microorganisms’ surfaces, exposing them to high local levels of cytotoxic molecules. In addition, experiments conducted on primary human neutrophils have shown that NETs indirectly augment the complement’s capacity to destroy pathogens [44]. Although NETs can protect the host from microbes, excessive production can be harmful, because they protract the inflammatory process and delay wound healing. Recent studies with in vitro experiments and animal models have shown that NETs play a crucial role in the pathogenesis of some metabolic, autoimmune, and auto inflammatory diseases, as well as in some septic conditions that increase morbidity and mortality [45]. Neutrophils are important in the wound repair process, not only for their ability to eliminate pathogens, but also for their ability to release substances that amplify the inflammatory process. Several studies have shown that activated neutrophils overexpress genes involved in the healing process. Specifically, activated neutrophils hyper-express cytokines and chemokines (TNF-α, IL-1β, IL-6, CXCL2, CXCL8, and VEGF-A) which promote inflammatory response, stimulate fibroblast and keratinocyte proliferation, and promote angiogenesis [46]. In a normal healing process, the neutrophils at the wound site undergo apoptosis after fulfilling their functions. Macrophages phagocytose apoptotic neutrophils, strongly signaling inflammatory resolution. In fact, excessive neutrophil activity and persistence at the wound site results in a prolonged inflammatory state and the development of a chronic wound [43] (Figure 2).

### 4.2. Macrophages

Macrophages are critical for wound healing and tissue regeneration. In intact skin, resident macrophages function as homeostatic sentinels.

#### 4.2.1. M1 Macrophages

In case of skin injury, PAMPs and DAMPs stimulate the polarization of resident macrophages into the M1 pro-inflammatory phenotype [47]. M1 macrophages, also known as “classically activated macrophages”, support the inflammatory process by releasing cytokines such as IL-1β, IL-12, TNF-α, IL-6, and IL-23, which prevent pathogen passage and alarm the adaptive immune system [48]. M1 macrophages can activate T lymphocytes due to the abundance of MHC class I and class II molecules, as well as CD80/CD86 costimulatory molecules, on their surface [47,49]. Furthermore, M1 macrophages prevent infection by phagocytosing pathogen phagosomes rich in reactive oxygen species (ROS) [50]. Proinflammatory macrophages also secrete metalloproteinases, including MMP9, which degrade the extracellular matrix and fibrin thrombus, thus promoting their migration. Digested ECM fragments act as immunostimulatory DAMPs, aggravating the proinflammatory state of the wound [51]. In addition to being bactericidal, macrophages also carry out efferocytosis, a multi-step process by activated macrophages aimed at removing apoptotic cells, such as neutrophils, after their internalization and digestion, which that is very important to eliminating neutrophils and resolving inflammation.

#### 4.2.2. M2 Macrophages

As the inflammatory process subsides, macrophages switch from an inflammatory phenotype to an M2 anti-inflammatory phenotype. M2 macrophages, also called “alternatively activated macrophages”, express anti-inflammatory mediators such as IL-10, TGF-β, VEGF-A, and IGF1. They also promote fibroblast proliferation, ECM synthesis, and angiogenesis [52]. The transition from M1 to M2 is crucial for resolving inflammation and shifting the balance toward tissue repair [53]. Neutrophil phagocytosis also facilitates this conversion. Indeed, the impairment of efferocytosis in diabetic wounds and aged mice prolongs the inflammatory phase and delays healing [54]. In the proliferative phase, M2 macrophages release TGF-β, VEGF-A and other cytokines that trigger the transition from fibroblast to myofibroblast [55]. Myofibroblasts release metallo-proteinases that degrade the provisional matrix and synthesize ECM components, such as fibronectin, collagen, and proteoglycans [1]. This process contributes to the formation of granulation tissue, which serves as a scaffold for wound cell migration and differentiation, in addition to supporting the formation of new blood vessels [56]. In contrast, certain inflammation mediators released by M1 macrophages, including TNF-α, inhibit differentiation, confirming that prolonged inflammation results in delayed wound closure [3]. In addition, cytokines released by M2 macrophages, such as PDFG, VEGF-A, and TGF-β, promote the formation of new capillaries in endothelial cells, which are needed to transport oxygen and nutrients, as well as to enable tissue regeneration [57]. Angiogenesis, the process of new blood vessel formation, is essential for successful wound healing [58]. After the re-epithelialization phase, macrophages regain their phagocytic phenotype and transform into a subgroup known as M2c. M2c macrophages release proteases, phagocytose cells, and matrix components that are no longer necessary for the healing process. Several pathologies are associated with altered macrophage activity. Delayed macrophage influx can result in delayed efferocytosis, resulting in accumulated neutrophils, extracellular matrix proteins, and debris in the wound, which can cause a chronic inflammatory state [3]. Conversely, excessive macrophage activity in the final stage of the repair process overstimulates fibroblasts, with subsequent accumulation of extracellular matrix proteins and formation of fibrotic scars [1].

### 4.3. Mast Cells

Mast cells (MCs) are immune system cells that originate from CD34+/CD117+ bone marrow progenitor cells. They are involved in inflammatory processes and allergic reactions through the release of mediators stored in cytoplasmic granules [59]. When an injury occurs, a variety of mediators, such as monocyte chemoattractant protein-1 (MCP-1), released from keratinocytes recruit mast cells to the injured site [60]. Once activated, mast cells release vasoactive and proinflammatory mediators. These include histamine, tryptase, TNF-α, VEGF-A, IL-6, and IL-8. Tryptase binds to protease-activated receptor 2 (PAR2) on endothelial cells and, together with histamine, induces vasodilation by promoting the infiltration of neutrophils and inflammation mediators at the site of injury [61]. Histamine also promotes the antibacterial activity of keratinocytes. This is achieved by enhancing the expression of pathogen recognition receptors, such as TLR-2, and increasing their GM-CSF production by promoting macrophage maturation. Additionally, IL-8, released by MCs, is involved in neutrophil chemotaxis. Tryptase, also released by MCs, promotes the migration and proliferation of fibroblasts and stimulates the release of ECM proteins [62]. Thus, mast cells release mediators that are relevant not only in the inflammatory phase, but also in the later stages of the wound healing process [63].

### 4.4. T-Cell Populations

#### 4.4.1. Th1 Lymphocytes

In the inflammatory phase of the healing process, cytokines released in the microenvironment recruit Th1 lymphocytes to the wound site, which play an important role in this phase. In fact, Th1 lymphocytes produce pro-inflammatory cytokines, such as IL-2, which acts as a survival signal for T-reg, T helper, and cytotoxic T cells, and IFN-γ [64]. Although IFN-γ has potential antifibrotic effects, it is also a proinflammatory cytokine. IFN-y knockout mice had reduced inflammation but increased fibrosis, highlighting that inflammation and fibrogenesis are not always correlated [65]. Th1 cells also support the inflammatory process by promoting macrophage polarization into the M1 phenotype. In turn, M1 releases cytokines that promote Th1 aggregation at the injury site [66]. As the inflammatory phase subsides, the activity of Th1 decreases, and a reparative response mediated by Th2 lymphocytes begins.

#### 4.4.2. Th2 Lymphocytes

Th2 lymphocytes produce cytokines, such as IL-4, IL-5, IL-13, and IL-10, that promote collagen production by fibroblasts. IL-13 and IL-4 are the main stimulators of ECM deposition through the activation of the intracellular JAK pathway. This pathway leads to the phosphorylation of STAT6 and the activation of genes that promote collagen synthesis (Table 1 and Tables S1–S3). In addition, cytokines IL-4 and IL-13 induce macrophage polarization towards the M2 reparative phenotype. Studies on Th1 and Th2 have shown that the transition from the type 1 inflammatory response to the type 2 reparative process is fundamental for wound healing. This is because some fibrosis is intrinsically needed to effectively resolve physiological wound healing [66].

#### 4.4.3. Th17 and Th22 Lymphocytes

Several studies have shown that IL-22, produced by two subgroups of T lymphocytes (Th17 and Th22), contributes greatly in repair processes. In particular, IL-22 stimulates the formation of new blood vessels, granulation tissue, and keratinocyte proliferation, promoting re-epithelialization [67]. Treating diabetic mice with IL-22 significantly accelerated diabetic wound closure to the levels of non-diabetic wounds. Specifically, IL-22 treatment was found to be associated with increased vascular density and granulation tissue formation similar to those of nondiabetic wounds [68].

#### 4.4.4. T-Reg Lymphocytes

Other immune cells, T-regs, are key players in reducing the inflammatory response during the healing process [69,70]. In human skin, T-regs are inclined to exhibit a memory phenotype, and they are localized in areas of the epidermis and dermis surrounding hair follicles. These cells release cytokines, such as IL-10 and TGF-β, that stimulate macrophage polarization into M2 and suppress the inflammatory response. Ablation of T-regs in mouse excisional wound models leads to increased levels of IFN-γ and proinflammatory macrophages, resulting in a prolonged type 1 inflammatory response and delayed wound closure [66]. A recent study in Foxp3-DTR transgenic mice, where T-regs are depleted following injection with diphtheria toxin, found that the healing process was slower in T-reg-depleted mice than in wild-type controls [9]. T-regs are also involved in the pathological fibrosis of keloids, because they have been shown in vitro to upregulate collagen expression by fibrocytes, which are myeloid-derived profibrotic cells. This increase in collagen expression is mediated through TGF-β production by Tregs.

#### 4.4.5. γδ T Lymphocytes

In mice, a population of epidermis-resident γδ T cells with dendritic morphology (DETC) are involved in the maintenance of skin homeostasis and the promotion of wound repair by releasing, upon activation, insulin-like growth factor-1 (IGF-1), granulocyte macrophage colony stimulating factor (GM-CSF), IL-17, IL-13, and several chemokines that sustain the survival of keratinocytes and promote leukocyte recruitment [71,72]. In mice, it has been demonstrated that keratinocyte-released IL-15 regulates the production of IGF-1 by γδ T cells. Specifically, blocking IL-15 leads to a reduction in IGF-1 secretion, which delays the healing process. Furthermore, the involvement of T γδs in the repair process is also mediated by the release of IL-17A, which promotes the release of epidermal antimicrobial peptides, such as β-defensins, and chemokines, such as CCL3, CCL4, and CCL5, which support the inflammatory process by recruiting immune cells [71]. Mice lacking γδ T cells show delayed wound closure due to reduced keratinocyte proliferation, delayed macrophage infiltration, and less deposition of ECM [73]. Although DECTs are absent in humans, human skin harbors a population of gd T cells, equipped with a limited TCR repertoire, whose antigen specificity and role in wound healing is still largely undefined [74].

### 4.5. Langerhans Cells and Dermal Dendritic Cells

Langerhans cells (LCs) are professional antigen-presenting cells of myeloid origin resident in the epidermis. LCs constitutively express major histocompatibility complex class I and II (MHCI/MHCII) molecules and present antigens to CD8+ and CD4+ T lymphocytes [75]. The LC membrane is rich in structures that allow adhesion with T cells or act as co-stimuli for T cell activation (CD80, CD86). Under homeostasis, Langerhans cells persist in the epidermis through interactions between epithelial cell adhesion molecules (EpCAMs), or E-cadherin expressed on LCs, and the E-cadherin exposed by keratinocytes. When exposed to mediators of inflammation and other activators, such as PAMPs, LCs migrate from the epidermis to the lymph nodes, where they activate the adaptive immune response by deactivating the adhesion interaction between E-cadherin and EpCAM [22]. In particular, the migration of LCs through the dermis is mediated by CXCR4 signaling after binding to the chemokine CXCL12, which is produced by dermal fibroblasts [76]. In injured skin, endogenous alarmins, cytokines produced by damaged cells, and other inflammatory signals, such as PAMPs, stimulate the recruitment, activation, and maturation of LCs. Activated LCs increase the expression of MHC-II molecules and promote T-cell activation [77]. LCs also express C-type lectin Langerin (CD207), which is involved in the formation of Birbeck granules.

The role of LCs in the wound repair process has not been widely explored, but several data suggest that LCs could inhibit proliferation in the epidermis. Increased LC density has been observed in chronic [78] and hypertrophic wounds [79]. Depletion of LCs promotes keratinocyte proliferation, granulation tissue, and new blood vessel formation in Langerin DTR transgenic mice. In fact, the wounds of Langerin DTR transgenic mice healed faster than those of the control group, indicating a suppressive role of LCs in regulating cutaneous wound healing [80]. Dermal dendritic cells (dDCs) migrate to lymph nodes and present antigen to T cells, similar to LCs. Dermal dendritic cells are classified into the following two main groups: conventional dendritic cell type one (cDC1) and conventional cell type two (cDC2). In contrast to murine cDC1, human dermal dendritic cells do not express Langerin [22]. The cDC1 (141+) co-expresses CD304 (neuropilin-1), XCR1, and CD370, and activates a Th1 immune response. On the other hand, cDC2 co-expresses CD11b and CX3CR1, and activates a Th2 response. Immature dermal dendritic cells express pattern recognition receptors, such as TLR2, TLR4, CD206, and CD209. In contrast, mature dermal dendritic cells express high levels of costimulatory molecules, such as CD83, and lower levels of pattern recognition receptors. Activated dermal dendritic cells participate in the inflammatory response by releasing cytokines, such as TNF-α, and chemokines that contribute to the elimination of infectious agents. However, in some cases, their activation underlies a pathological tissue response with persistent inflammation [81]. Other dendritic cells are plasmacytoid dendritic cells (pDCs), which are present in the skin exclusively under inflammatory conditions [82]. The pDCs produce high amounts of INF-α. Depletion of pDCs in mice treated with bleomycin, which induces skin fibrosis, showed a decrease in skin thickness and collagen content compared to those in wild-type mice, suggesting a role for pDCs in fibrosis [22]. Further studies are required to better define the role of DCs in the wound repair process.

## 5. Role of Immune Cells in the Remodeling Phases of Wound Healing

Remodeling represents the last stage of the wound repair process and is characterized by the regression of angiogenesis, the replacement of type III collagen with type I collagen, and the transition of granulation tissue into scar tissue. In addition, most of the cells involved in the previous stages undergo apoptosis [57]. The main cells involved in this phase are myofibroblasts and macrophages, which release MMPs promoting ECM reorganization. In addition, macrophages present at the wound site assume a fibrolytic phenotype, degrading excess ECM fragments and phagocytosing dead cells and debris. In a normal repair process, at the end of the remodeling phase, myofibroblasts also undergo apoptosis and are phagocytized by macrophages. Missed apoptosis of cells in granulation tissue leads to the development of hypertrophic scars [3].

## 6. Conclusions and Future Directions

Cells of the innate and adaptive immune system cooperate with resident cells to provide integrated and sometimes redundant mechanisms to ensure proper wound repair. Dysregulation of immune cell activity at any stage of the process can lead to pathological conditions such as chronic wounds, hypertrophic wounds, or keloids. Chronic wounds, to date, represent an important cause of morbidity and mortality and are of serious clinical concern. In fact, a characteristic feature of chronic wounds is the presence of a chronic inflammatory process, due to a prolonged presence of pro-inflammatory cells in the wound. In chronic or hypertrophic wounds, there is dysregulation in the production of pro-inflammatory factors, such as TNF-α, IL-1, or IL-6, or anti-inflammatory factors, such as IL-4, IL-10, or TGF-β. These cytokines, therefore, represent important therapeutic targets, so strategies that modulate the immune system may be an innovative therapeutic approach for the treatment or prevention of chronic wounds. However, it must be considered that modulation of the immune system may have negative effects at sites other than the wound, or on other patient conditions, such as infections or allergies. Therefore, given the high complexity of the healing process, it is essential to conduct further studies to better understand the mechanisms underlying both normal and pathological healing.

## Figures and Tables

**Table 1:** The main signaling pathways required in different phases of wound healing.

Wound Healing Phase	Cell Type	Cytokines	Signaling Pathways
** INFLAMMATION **	NEUTROPHILES	TNF-α IL-1β IL-6 VEGF	NF-kB NF-kB STAT3 ERK
	M1 MACROPHAGES	TNF-α IL-1β IL-6 IL-12 IL-23	NF-kB NF-kB STAT3 STAT4 STAT4
	MAST CELL	TNF-α IL-6 VEGF IL-8	NF-kB STAT3 ERK STAT3/ERK
	TH1 LYMPHOCYTES	INF-γ IL-2 IL-12	STAT1 STAT3/ERK STAT4
** PROLIFERATION **	M2 MACROPHAGES	IL-10 TGF-β VEGF IGF1	STAT3 SMAD ERK ERK
	TH2 LYMPHOCYTES	IL-4 IL-5 IL-13	STAT6 ERK/NF-kB STAT6
** REMODELING **	T-REG	IL-10 TGF-β	STAT3 SMAD

## Supplementary Materials

The following supporting information can be downloaded at: https://www.mdpi.com/article/10.3390/cells13070624/s1, Table S1: GlyGly (K) Sites; Table S2: Quantitative and differential analysis; Table S3: DiGly sites exclusively identified in one group.
